# Durvalumab after chemoradiotherapy for locally advanced non-small cell lung cancer prolonged distant metastasis-free survival, progression-free survival and overall survival in clinical practice

**DOI:** 10.1186/s12885-022-09354-1

**Published:** 2022-04-04

**Authors:** Takaya Yamamoto, Yoko Tsukita, Yu Katagiri, Haruo Matsushita, Rei Umezawa, Yojiro Ishikawa, Noriyoshi Takahashi, Yu Suzuki, Kazuya Takeda, Eisaku Miyauchi, Ryota Saito, Yoshiyuki Katsuta, Noriyuki Kadoya, Keiichi Jingu

**Affiliations:** 1 Department of Radiation Oncology, Tohoku University Graduate School of Medicine, Sendai, Japan; 2 Department of Respiratory Medicine, Tohoku University Graduate School of Medicine, Sendai, Japan

**Keywords:** Intensity-modulated radiotherapy, IMRT, Chemoradiotherapy, Durvalumab, Time-dependent covariate

## Abstract

**Background:**

In clinical practice, the effect of durvalumab and radiation pneumonitis (RP) on survival after intensity-modulated radiotherapy (IMRT) is not fully understood. The purpose of this retrospective study was to investigate factors related to distant metastasis-free survival (DMFS), progression-free survival (PFS) and overall survival (OS) after IMRT for locally advanced non-small cell lung cancer (LA-NSCLC).

**Methods:**

All patients who were treated with conventional fractionated IMRT for LA-NSCLC between April 2016 and March 2021 were eligible. Time-to-event data were assessed by using the Kaplan–Meier estimator, and the Cox proportional hazards model was used for prognostic factor analyses. Factors that emerged after the start of IMRT, such as durvalumab administration or the development of RP, were analysed as time-dependent covariates.

**Results:**

A total of 68 consecutive patients treated with conventional fractionated IMRT for LA-NSCLC were analysed. Sixty-six patients completed radiotherapy, 50 patients received concurrent chemotherapy, and 36 patients received adjuvant durvalumab. During the median follow-up period of 14.3 months, 23 patients died, and tumour progression occurred in 37 patients, including 28 patients with distant metastases. The 1-year DMFS rate, PFS rate and OS rate were 59.9%, 48.7% and 84.2%, respectively. Grade 2 RP occurred in 16 patients, grade 3 in 6 patients and grade 5 in 1 patient. The 1-year cumulative incidences of grade 2 or higher RP and grade 3 or higher RP were 33.8% and 10.3%, respectively. The results of multivariate analyses showed that durvalumab had a significantly lower hazard ratio (HR) for DMFS, PFS and OS (HR 0.31, *p* < 0.01; HR 0.33, *p* < 0.01 and HR 0.32, *p* = 0.02), respectively. Grade 2 or higher RP showed significance for DMFS and a nonsignificant trend for OS (HR 2.28, *p* = 0.04 and HR 2.12, *p* = 0.13), respectively, whereas a higher percentage of lung volume receiving 20 Gy or higher was significant for PFS (HR 2.25, *p* = 0.01).

**Conclusions:**

In clinical practice, durvalumab administration following IMRT with concomitant chemotherapy showed a significant survival benefit. Reducing the risk of grade 2 or higher RP would also be beneficial.

## Background

Definitive-intent radiotherapy with or without chemotherapy has been a standard treatment for patients with locally advanced non-small cell lung cancer (LA-NSCLC) who are unresectable or inoperable but are treatable with radiotherapy [1, 2]. If patients can tolerate it, chemoradiotherapy is superior to radiotherapy alone, and the 2-year survival rate increases from 21.4% to 25.4% by adding chemotherapy to radiotherapy [3]. Recently, a remarkable survival benefit was shown in a randomized phase III trial, the so-called PACIFIC study, by combining chemoradiotherapy with an immune checkpoint inhibitor, durvalumab, which blocks programmed death ligand 1 (PD-L1) [4, 5]. By using durvalumab after chemoradiotherapy for LA-NSCLC, these patients had significantly longer distant metastasis-free survival (DMFS), progression-free survival (PFS) and overall survival (OS) than patients treated with placebo. The PACIFIC study showed impressive outcomes, but the benefit of this treatment approach in the real world and in clinical practice has not been fully evaluated [6].

In addition to attracting attention to durvalumab, radiotherapy has also been refocused, partly because durvalumab needs to be discontinued if there is grade 2 or higher radiation pneumonitis (RP). Among the various radiotherapy techniques, intensity-modulated radiotherapy (IMRT) has a lower rate of grade 2 and 3 RP than three-dimensional conformal radiotherapy and possibly a longer survival [7–9]. Risk factors for grade 2 or higher RP after IMRT in this durvalumab era have been reported [10]. However, the effect of RP on survival has not been fully elucidated, especially after the emergence of durvalumab. Furthermore, the benefit of durvalumab for patients who are indicated for definitive-intent IMRT has not been fully investigated because, in the PACIFIC study, the radiotherapy techniques included both IMRT and three-dimensional conformal radiotherapy, and the patients were randomized after chemoradiotherapy.

In clinical practice, patients who have an indication for concurrent chemoradiotherapy might receive only definitive-dose radiotherapy because of infection, such as obstructive pneumonitis, or patient refusal. There are patients who refuse the administration of durvalumab, or who develop RP before or immediately after starting durvalumab. Because subgroup analyses of the PACIFIC study suggested that a shorter interval between the last radiation and randomization (less than 14 days) showed greater effectiveness of durvalumab, pulmonologists tend to rush to administer durvalumab, possibly resulting in the development of RP immediately after the initiation of durvalumab [4]. This tendency might affect outcomes in clinical practice because the subgroup with a shorter interval accounts for only approximately 25% in the PACIFIC study. Therefore, assessing the impacts of concurrent chemotherapy, durvalumab and RP in the real world is important.

To assess the effects of factors that appear after the start of radiotherapy, it is useful to analyse these factors as time-dependent covariates in a Cox model [11]. By using a Cox proposal hazard model with time-dependent covariates, it is possible to assess the impact of durvalumab and RP on survival because these factors appear after the completion of radiotherapy. This study aimed to investigate the effect of both pretreatment factors and factors that emerged after the start of radiotherapy, such as durvalumab and RP, on OS, PFS and DMFS.

## Patients and methods

### Eligibility criteria

Data were obtained from our clinical database. Patients who were treated with definitive-intent conventional fractionated IMRT for LA-NSCLC between April 2016 and March 2021 at Tohoku University Hospital were identified. Adjuvant radiotherapy after surgery or radiotherapy for postoperative recurrence was not included. All identified patients were included in this study regardless of completion of radiotherapy, administration of concurrent chemotherapy or the follow-up period. Data from a total of 68 patients were analysed. The date of data cut-off was October 31, 2021.

### Pretreatment characteristics and outcome assessments

The patient, lung cancer and treatment characteristics are shown in Table 1. Four patients had an ECOG performance status (PS) of 2 at the start of radiotherapy. Nine patients were never smokers, and the median Brinkman index (the number of cigarettes smoked per day × the number of years of the habit) was 800 (range, 0–1900). Patients who had been diagnosed with interstitial lung disease by pulmonologists at the time of the start of radiotherapy were classified into the interstitial lung disease group. The stage of each cancer was based on the 8th edition of the UICC classification. Two patients were diagnosed with stage IVA: 1 patient had two ipsilateral axillary node metastases, and the other had single pleural dissemination. Both patients received definitive-dose IMRT for all lesions. All patients received involved field IMRT using volumetric modulated arc therapy (VMAT).

**Table 1:** Patient, lung cancer and treatment characteristics

Category	Variables	68 patients (%)
Age, years	Median (range)	71 (43–84)
Sex	Female	13 (19.1)
	Male	55 (80.8)
ECOG PS	0	37 (54.4)
	1	27 (39.7)
	2	4 (5.8)
Charlson comorbidity index	0	25 (36.7)
	1	23 (33.8)
	2	15 (22.0)
	3–4	5 (7.3)
Smoking status	Never	9 (13.2)
	Current	27 (39.7)
	Former	32 (47.0)
Brinkman index	Median (range)	800 (0–1900)
FEV1 (L)	Median (range)	2.18 (1.12–3.67)
FEV1 (% of predicted)	Median (range)	88.0 (36.4–125.4)
FEV1/FVC (%)	Median (range)	71.6 (32.1–89.0)
Interstitial lung disease	No	65 (95.5)
	Yes	3 (4.4)
Diabetes mellitus	No	56 (82.3)
	Yes	12 (17.6)
COPD	No	48 (70.5)
	Yes	20 (29.4)
Pathology	Adenocarcinoma	32 (47.0)
	Squamous cell carcinoma	27(39.7)
	Others	9 (13.2)
PD-L1 tumour proportion score	< 1%	11 (16.2)
	≥ 1%	31 (45.6)
	Unknown	26 (38.2)
Stage (UICC 8th)	IIA-IIB	8 (11.7)
	IIIA	23 (33.8)
	IIIB	31 (45.5)
	IIIC-IVA	6 (8.8)
Chemotherapy	No	18 (26.4)
	Yes	50 (73.5)
	- Cisplatin/vinorelbine	24
	- Carboplatin/paclitaxel or nab-paclitaxel	20
	- Others	6
Total prescribed dose	60 (Gy)	56 (83.3)
	66 (Gy)	10 (14.7)
	Others	2 (2.9)
PTV (cc)	Median (range)	305.5 (77.6–958.9)
Radiation dose parameters	Dose coverage 90% of CTV (Gy)	60.9 (58.8–68.6)
	Dose coverage 90% of PTV (Gy)	58.9 (55.5–66.0)
Median (range)	Lung V5 Gy (%)	56.2 (27.9–84.0)
	Lung V20 Gy (%)	23.6 (11.1–35.1)

*ECOG* Eastern Cooperative Oncology Group, *PS* performance status, *FEC* forced expiratory volume, *FVC* forced vital capacity, *COPD* chronic obstructive pulmonary disease, *PD-L1* programmed death-ligand 1, *PTV* planning target volume, *CTV* clinical target volume, *Lung Vn Gy* percentage of total lung volume exceeding *n* *Gy*

DMFS was defined as the interval from the start of radiotherapy to the date that distant metastasis or death was confirmed. PFS was defined as the interval from the start of radiotherapy to the date of any tumour progression or death. OS was defined as the interval from the start of radiotherapy to the date of death from any cause. The tumour response to radiotherapy and tumour progression were judged according to the Response Evaluation Criteria in Solid Tumours (RECIST) version 1.1. Lung toxicities were judged according to the Common Terminology Criteria for Adverse Events version 5.0 (CTCAE v5.0).

### CT simulation and IMRT (VMAT) procedure

Each patient was immobilized in the supine position with a vacuum cushion (VacQfix Cushion, Qfix, Avondale PA, USA). If respiratory motion control was needed, oxygen inhalation and/or an abdominal pressure belt were used. Then, CT scans at intervals of 2.0–2.5 mm were performed for radiation planning and a 4-dimensional CT scan of the whole lung was performed for the measurement of intrafractional respiratory movement. Based on FDG-PET and the diagnostic CT images, gross tumour volume (GTV) was carefully contoured using the planning CT image. Then, the internal GTV was created using a 4-D CT image. The clinical target volume (CTV) expanded the internal GTV by 5 mm, and no prophylactic lymph node area was added, i.e., the involved field approach. A planning target volume (PTV) margin of 5 mm was added for setup and interfractional uncertainty. The treatment dose was prescribed covering 50%-90% of the PTV using the VMAT technique. Multiarc beams with 6 MV or 10 MV photons were used (Clinac 23EX or TrueBeam STx, Varian Medical Systems, Palo Alto, CA, or Versa HD, Elekta, Stockholm, Sweden). In principle, our main dose constraints were as follows: the percentage of total lung volume (lung minus GTV) exceeding 20 Gy (V20) and 5 Gy (V5) was less than 37% and 65%, respectively; the maximum dose of the spinal cord and spinal cord plus 3 mm did not exceed 46 Gy and 48 Gy, respectively; and less than 1% and 20% of the heart received 63 Gy and 50 Gy, respectively.

### Statistical analyses

Time-to-event data were assessed by using the Kaplan–Meier estimator. When the cumulative incidence of RP was calculated, death without RP was regarded as a competing risk. Continuous covariates were divided at the sample median into two groups. To perform multivariate risk factor analyses, we assumed that selection of the variables was needed because the sample size was limited and at least 5 events per variable were needed for the multivariate analyses (MVA) [12]. First, the Cox proportional hazards model was applied for each variable in univariate analyses (UVA) to measure the size of the effect on survival. Tumour response to radiotherapy, durvalumab administration, progression of irradiated sites, RP and steroid administration were regarded as time-dependent covariates, and Mantel-Byar tests were performed. Second, variables that showed *p* values of 0.10 or less than 0.10 from UVA were selected to create a plausible model for stepwise selection. Third, stepwise multivariate Cox proportional hazards models using the Akaike information criterion were performed to identify independent risk factors. Finally, a *p*-value less than 0.05 was defined as significant. Age and sex are well-known confounding factors; therefore, age and sex were used as stratification factors. In addition, age and sex were included in the MVA model as sensitivity analyses because age and sex were possible prognostic factors. Statistical analyses were performed using EZR version 1.54 (Saitama Medical Center, Jichi Medical University, Saitama, Japan), a modified version of R commander (R Foundation for Statistical Computing, Vienna, Austria) [13].

## Results

A total of 68 patients were analysed, and 2 patients (2.9%) did not complete the planned radiotherapy: 1 patient stopped at 58 Gy of 60 Gy because of febrile neutropenia, and another stopped at 60 Gy of 66 Gy because of RP or pneumonia. None of the patients received induction chemotherapy, and 50 patients (73.5%) received radiotherapy with concomitant chemotherapy. Thirty-six patients received durvalumab, and the mean and median intervals between the last radiotherapy and initiation of durvalumab were 5.5 days and 9.1 days, respectively (range, 1.0–36.0 days). The response to radiotherapy was a partial response in 24 patients, stable disease in 42 patients and progressive disease in 2 patients.

**Fig. 1:**
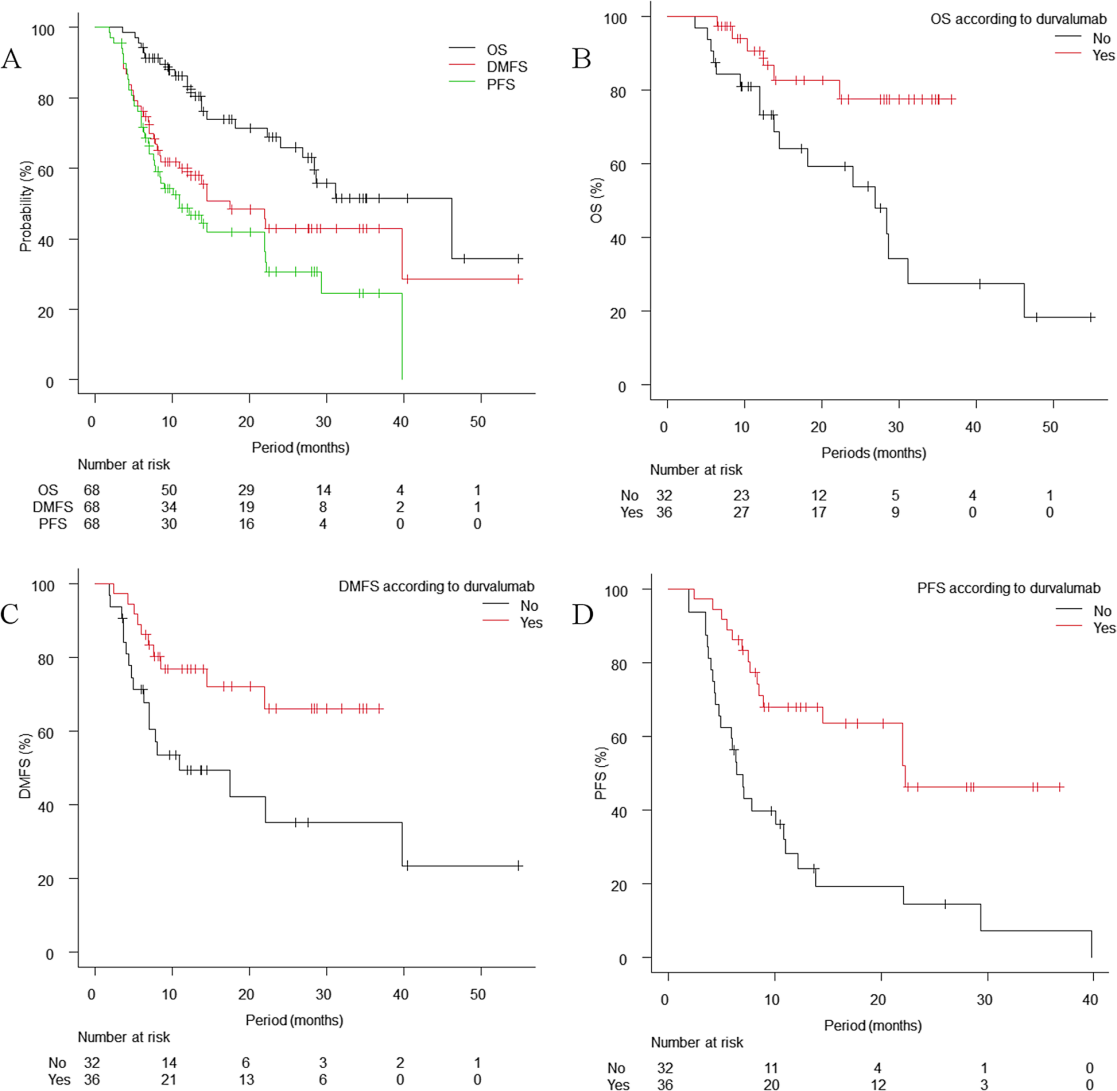
Kaplan–Meier curves of overall survival (OS), distant metastasis-free survival (DMFS)and progression-free survival (PFS). The 1-year DMFS rate, PFS rate and OS rate were 59.9%, 48.7% and 84.2%, respectively (**A**). OS, DMFS and PFS curves according to durvalumab administration are shown in **B**-**D**

**Fig. 2:**
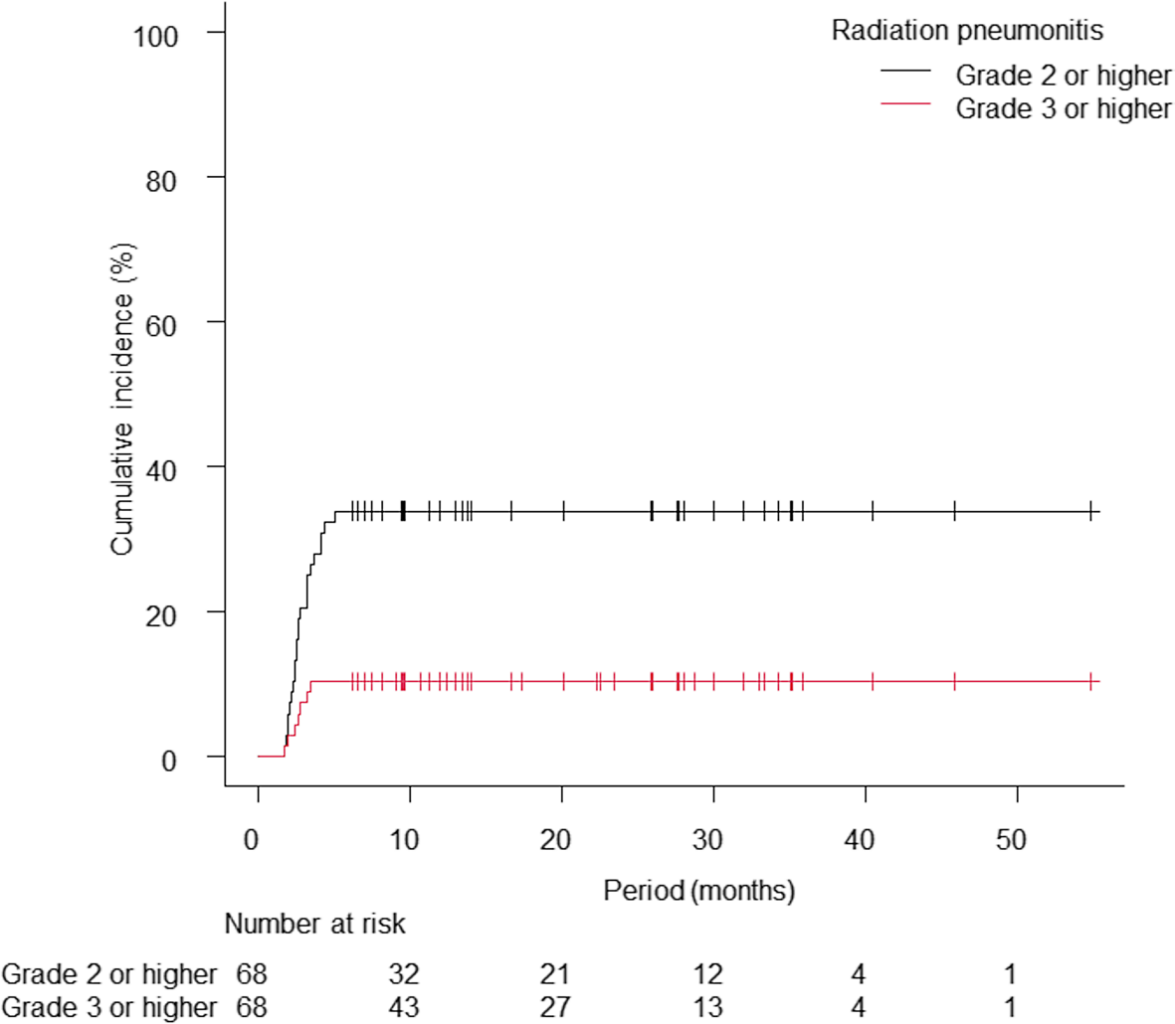
The cumulative incidence of grade 2 or higher radiation pneumonitis (RP) and grade 3 or higher RP are shown. Death due to RP was regarded as a competing risk, and the curve of the competing risk is not shown. The 1-year cumulative incidences of grade 2 or higher RP and grade 3 or higher RP were 33.8% and 10.3%, respectively

The median follow-up period for all patients was 14.3 months (range: 3.5–54.8 months) and that for survivors was 17.7 months (range: 6.2–54.8 months). During the follow-up, 23 patients died, and 17 of these died from lung cancer. Tumour progression occurred in 37 patients, including 16 patients with progression at irradiated sites and 28 patients with distant metastases. The first metastatic site was bone in 11 patients, brain in 9 patients and lung (lung metastases or dissemination) in 9 patients. The 1-year DMFS rate, PFS rate and OS rate were 59.9% (95% confidence interval [CI]: 46.8–70.7%), 48.7% (95% CI: 35.8–60.3%) and 84.2% (95% CI: 72.5–91.2%), respectively (Fig. 1A). The Kaplan–Meier curves according to durvalumab administration are shown in Fig. 1B, C and D. Regarding the toxicity of radiotherapy, grade 2 or higher RP occurred in 23 patients, and grade 3 or higher RP occurred in 7 patients, including 1 patient with grade 5 RP. The median time from the start of radiotherapy to the development of grade 2 or higher RP was 2.6 months (range, 1.7–5.1 months). Eleven patients who received durvalumab developed grade 2 or higher RP. Among the 3 patients with interstitial lung disease, 1 patient developed grade 2 RP, and 2 patients developed grade 3 RP, which occurred within 3 months of the start of radiotherapy. Steroids were administered to 29 patients excluding palliative care intent: 3 patients for comorbidities, 22 patients for RP and 4 patients for drug-induced toxicities. Two patients received steroid pulse therapy for RP, and one of them developed grade 5 RP or pneumonia. The 1-year cumulative incidences of grade 2 or higher RP and grade 3 or higher RP were 33.8% (95% CI: 22.8–45.1%) and 10.3% (95% CI: 4.5–18.9%), respectively (Fig. 2).

The results of UVA are summarized in Table 2, and the results of MVA are shown in the upper column of Table 3. The variables that were candidates for the MVA model but are not listed in Table 3 were removed from the MVA model as a result of stepwise selection. In MVA, durvalumab showed consistent significance with a low hazard ratio (HR) for DMFS, PFS and OS (HR 0.31, 95% CI 0.14–0.69, *p* < 0.01, HR 0.33, 95% CI 0.15–0.69, *p* < 0.01 and HR 0.32, 95% CI 0.12–0.86, *p* = 0.02), respectively. Grade 2 or higher RP showed significance in DMFS and a nonsignificant trend in OS (HR 2.28, 95% CI 1.02–5.10, *p* = 0.04 and HR 2.12, 95% CI 0.80–5.60, *p* = 0.13), respectively. Regarding PFS, grade 2 or higher RP was not selected as a prognostic factor, but instead, lung V20 showed significance (HR 2.25, 95% CI 1.13–4.45, *p* = 0.01). By applying sensitivity analyses, the significance of these factors was confirmed (Table 3, lower column). To evaluate the potential effect of stage and PD-L1 tumour proportion score, subgroups of stage III patients and patients administered durvalumab were analysed. There were no significant differences between stage IIIA and IIIB-IIIC in DMFS, PFS and OS (HR 1.08, *p* = 0.83, HR 0.97, *p* = 0.93 and HR 1.11, *p* = 0.82), respectively. Among patients administered durvalumab, the difference between PD-L1 ≥ 1% and PD-L1 < 1% was not significant for DMFS, PFS and OS (HR 0.44, *p* = 0.35, HR 0.66, *p* = 0.61 and HR 0.46, *p* = 0.51), respectively.

**Table 2:** The results of univariate analyses using the Cox proportional hazard model

Category, variable (reference)	Distant metastasis-free survival		Progression-free survival		Overall survival	
	HR (95% CI)	*P* value	HR (95% CI)	*P* value	HR (95% CI)	*P* value
Age, > 71 (≤ 71)	1.21 (0.61–2.38)	0.57	1.09 (0.59–2.02)	0.77	2.31 (0.96–5.56)	0.05
Sex, Male (Female)	0.88 (0.39–1.96)	0.76	1.17 (0.53–2.5)	0.68	1.77 (0.59–5.27)	0.30
ECOG PS 1–2 (0)	2.08 (1.04–4.16)	0.03	1.96 (1.05–3.64)	0.03	1.67 (0.72–3.82)	0.22
Charlson comorbidity index, 2–4 (0–1)	0.74 (0.34–1.59)	0.44	0.90 (0.46–1.75)	0.77	1.30 (0.54–3.11)	0.55
Brinkman index, > 800 (≤ 800)	1.25 (0.63–2.48)	0.50	0.89 (0.47–1.65)	0.71	2.41 (1.03–5.60)	0.04
FEV1 (L), > 2.2 (≤ 2.2)	0.80 (0.40–1.61)	0.54	0.82 (0.43–1.54)	0.54	0.72 (0.31–1.71)	0.46
FEV1 (% of predicted), > 88 (≤ 88)	0.97 (0.48–1.95)	0.94	0.85 (0.45–1.60)	0.61	0.48 (0.20–1.17)	0.10
FEV1/FVC (%), > 71 (≤ 71)	1.13 (0.56–2.27)	0.72	1.06 (0.56–2.00)	0.83	0.68 (0.29–1.60)	0.38
Interstitial lung disease, No (Yes)	1.55 (0.36–6.55)	0.55	1.42 (0.33–6.00)	0.62	4.58 (0.98–21.34)	0.05
Diabetes mellitus, No (Yes)	1.46 (0.63–3.40)	0.37	1.31 (0.60–2.88)	0.48	2.00 (0.72–5.54)	0.17
COPD, No (Yes)	0.97 (0.46–2.06)	0.95	0.78 (0.38–1.61)	0.50	1.21 (0.51–2.87)	0.65
Pathology, Adenocarcinoma (Others)	0.54 (0.26–1.12)	0.10	0.73 (0.39–1.38)	0.34	0.30 (0.11–0.84)	0.02
PD-L1 tumour proportion score, ≥ 1% (< 1%)	0.63 (0.35–1.58)	0.33	0.83 (0.34–1.99)	0.68	0.65 (0.21–2.02)	0.46
Stage, IIA-IIIA (IIIB-VIA)	0.68 (0.34–1.35)	0.27	0.69 (0.37–1.28)	0.25	0.66 (0.28–1.52)	0.33
Chemotherapy, Yes (No)	0.42 (0.20–0.86)	0.01	0.46 (0.24–0.89)	0.02	0.40 (0.16–0.98)	0.04
PTV (cc), > 305 (≤ 305)	0.98 (0.49–1.96)	0.97	1.00 (0.54–1.86)	0.99	1.59 (0.69–3.66)	0.26
Dose coverage 90% of CTV (Gy), > 60.9 (≤ 60.9)	1.39 (0.70–2.73)	0.33	1.21 (0.65–2.25)	0.53	0.91 (0.39–2.07)	0.82
Dose coverage 90% of PTV (Gy), > 59.0 (≤ 59.0)	1.08 (0.55–2.13)	0.81	1.05 (0.56–1.94)	0.87	0.77 (0.33–1.79)	0.55
Lung V5 Gy (%), > 56.2 (≤ 56.2)	0.92 (0.47–1.81)	0.82	1.44 (0.77–2.69)	0.24	0.79 (0.34–1.81)	0.58
Lung V20 Gy (%), > 23.6 (≤ 23.6)	1.59 (0.80–3.14)	0.18	2.28 (1.21–4.29)	0.01	1.44 (0.63–3.32)	0.38
Overall treatment period of RT (days), 42–45 (46–59)	1.81 (0.90–3.63)	0.09	1.70 (0.91–3.18)	0.09	1.50 (0.64–3.48)	0.34
Response to RT, PR (SD or PD)	0.51 (0.23–1.12)	0.09	0.57 (0.29–1.14)	0.11	0.30 (0.10–0.89)	0.03
Durvalumab administration, Yes (No)	0.33 (0.16–0.69)	< 0.01	0.32 (0.16–0.61)	< 0.01	0.28 (0.11–0.74)	< 0.01
Progression of irradiated sites, Yes (No)	1.85 (0.63–5.37)	0.25	-	-	1.54 (0.57–4.13)	0.38
Steroid administration, Yes (No)	1.72 (0.85–3.47)	0.12	1.26 (0.66–2.41)	0.46	2.26 (0.95–5.34)	0.06
Grade 2 or higher RP, Yes (No)	1.96 (0.96–4.03)	0.06	1.52 (0.78–2.95)	0.20	2.14 (0.92–5.01)	0.07
Grade 3 or higher RP, Yes (No)	1.40 (0.42–4.64)	0.58	1.65 (0.58–4.72)	0.34	2.21 (0.64–7.52)	0.20

*ECOG* Eastern Cooperative Oncology Group, *PS* performance status, *FEC* forced expiratory volume, *FVC* forced vital capacity, *COPD* chronic obstructive pulmonary disease, *PD-L1* programmed death-ligand 1, *PTV* planning target volume, *CTV* clinical target volume, *Lung Vn Gy* percentage of total lung volume exceeding *n* Gy, *RT* radiotherapy, *PR* partial response, *SD* stable disease, *PD* progressive disease, *RP* radiation pneumonitis

**Table 3:** The results of multivariate analyses using the Cox proportional hazard model stratified by age and sex (upper column) and multivariate analyses with inclusion of age and sex (lower column)

Category, variable (reference)	Distant metastasis-free survival		Progression-free survival		Overall survival	
	HR (95% CI)	*P* value	HR (95% CI)	*P* value	HR (95% CI)	*P* value
Lung V20 Gy (%), > 23.6 (≤ 23.6)	NS		2.25 (1.13–4.45)	0.01	NS	
Durvalumab administration, Yes (No)	0.31 (0.14–0.69)	< 0.01	0.33 (0.15–0.69)	< 0.01	0.32 (0.12–0.86)	0.02
Grade 2 or higher RP, Yes (No)	2.28 (1.02–5.10)	0.04	NS		2.12 (0.80–5.60)	0.13
Age, > 71 (≤ 71)	0.99 (0.48–2.04)	0.98	1.04 (0.55–1.97)	0.89	2.19 (0.88–5.44)	0.09
Sex, Male (Female)	0.63 (0.26–1.48)	0.29	0.80 (0.35–1.80)	0.59	1.13 (0.35–3.60)	0.83
Lung V20 Gy (%), > 23.6 (≤ 23.6)	NS		2.26 (1.18–4.33)	0.01	NS	
Durvalumab administration, Yes (No)	0.30 (0.14–0.64)	< 0.01	0.33 (0.17–0.65)	< 0.01	0.24 (0.09–0.66)	< 0.01
Grade 2 or higher RP, Yes (No)	2.31 (1.05–5.09)	0.03	NS		2.19 (0.88–5.44)	0.09

*NS* not selected because of the result of univariate analyses, *Lung Vn Gy* percentage of total lung volume exceeding *n* Gy, *RP* radiation pneumonitis

## Discussion

The current study showed the clinical outcomes of IMRT for LA-NSCLC and analysed not only pretreatment factors but also factors that emerged during the course of the treatment. The survival benefit of durvalumab was confirmed in clinical practice. The PACIFIC study revealed that durvalumab prolonged DMFS, PFS and OS, and its survival superiority seemed to be stable for a relatively long time [4, 5, 14]. In clinical practice, patients who did not fulfil the eligibility criteria of clinical trials could also be administered chemotherapy and durvalumab, leading to worse outcomes, as expected from the results of the clinical trial [6, 15, 16]. Fortunately, a recent real-world prospective study showed the safety profile of durvalumab maintenance treatment and comparable PFS with the PACIFIC study [17]. The current study also showed consistent and significant benefits of durvalumab in terms of DMFS, PFS and OS. Aside from the effectiveness and safety of durvalumab, there are other reasons. First, because the pulmonologists decided to administer concurrent chemotherapy and adjuvant durvalumab, there must be confounding by indication that did not emerge as a significant factor. Second, the compliance with IMRT was relatively good. For example, the ranges of lung V5 and V20 of the former prospective phase III trial (RTOG 0617) were 5.6–97.9% and 0.0–71.6%, respectively, in contrast to those of this study of 27.9–84.0% and 11.1–35.1%, respectively [18]. Finally, there was pressure for pulmonologists to rush to administer durvalumab after IMRT because of the subgroup analyses of the PACIFIC study in which patients with a shorter interval between the last radiation and randomization showed a lower HR, and the short interval in this study worked well, as expected from the subgroup analyses [4]. Overall, durvalumab showed a significant benefit for DMFS, PFS and OS in clinical practice.

Grade 2 or higher RP was a significant unfavourable factor for DMFS and it had a nonsignificant trend in OS. Regarding PFS, a higher lung V20 was a significant unfavourable factor, and a factor of grade 2 or higher RP was not selected. However, grade 2 or higher RP and lung V20 would have relationships because lung V20 is one of the representative risk factors for grade 2 or higher RP, and the importance of lung V20 is evident in this durvalumab era [19–23]. This result is therefore thought to be reasonable, and it is important to reduce lung doses, such as V20, to decrease the risk of developing grade 2 or higher RP. One of the reasons why grade 2 or higher RP had a significantly higher HR for DMFS and a tendency of a higher HR for OS is related to the administration of durvalumab. When patients develop grade 2 or higher RP, initiation of durvalumab is postponed or discontinued, leading to no administration of durvalumab or a smaller total dose (cycles) of durvalumab. Unfortunately, the total dose (cycles) of durvalumab was not analysed in this study because there were patients who have not yet completed 1-year treatment with durvalumab, and this issue will be addressed further in future works.

Interstitial lung disease, usually excluded from clinical trials, shows a high incidence of grade 2 or higher RP. Although only 3 patients in this study had interstitial lung disease, all of them developed grade 2 or higher RP, including 2 patients who developed grade 3 RP. Kobayashi et al. reported that 17 out of 37 patients with interstitial lung disease who received curative-intent chemoradiotherapy developed a grade 3 or worse acute exacerbation of interstitial lung disease [24]. In stereotactic lung radiotherapy, Onishi et al. reported that among 242 patients with pretreatment pulmonary interstitial changes, 6.9% developed fatal RP, and this rate is very high considering the 0.6% of grade 5 complications from Japanese survey data [25, 26]. In a recent meta-analysis, the treatment-related mortality of stereotactic radiotherapy was 15.6% in patients with interstitial lung disease [27]. Furthermore, patients with interstitial lung disease showed a higher incidence of severe immune checkpoint inhibitor-related pneumonitis [28]. Fortunately, grade 4–5 RP did not occur in any patients with interstitial lung disease in this study. Only patients who had been diagnosed with interstitial lung disease by pulmonologists at the time of starting radiotherapy were classified as interstitial lung disease in this study, but it might be more useful and sensitive to severe RP to assess the pretreatment pulmonary interstitial changes on CT images considering the report from Onishi et al. Great care should be taken when treating LA-NSCLC patients with interstitial lung disease and the appropriate degree of consideration should be taken when deciding whether to perform radiotherapy on these patients.

Various prognostic factors derived from pretreatment or treatment characteristics have been reported. Saunders et al. reported that hyperfractionated accelerated radiotherapy (56 Gy in 36 fractions of 1.5 Gy 3 times per day) showed 22% and 21% reductions in the relative risk of death and local progression, respectively [29]. They found that improved local tumour control can reduce the incidence of metastasis and that control of local tumours can lead to an improvement in long-term survival. However, dose escalation from 60 to 74 Gy failed to show a survival advantage, and it even resulted in a shorter survival of patients who received 74 Gy radiotherapy [18, 30]. Schild et al. also reported that concurrent chemoradiotherapy and involved field radiotherapy were associated with better survival in LA-NSCLC, and radiation dose levels did not show a significant association with survival [31]. Ostheimer et al. reported that absolute volume reduction of GTV from before chemoradiotherapy to during chemoradiotherapy at 40–50 Gy was correlated with survival, but only GTV of pretreatment was not significantly associated with survival [32]. PTV was also reported to be a prognostic factor. A larger PTV was significantly related to a higher HR of OS in MVA, and the difference was confirmed after propensity-matched analyses [33]. Katagiri et al. reported that adenocarcinoma pathology had a longer survival than squamous cell carcinoma pathology [34].

In this immune checkpoint inhibitor era, prognostic factors might be changed or strengthened. Above all, the effect of durvalumab was very strong. Taugner et al. analysed patients treated with chemoradiotherapy and durvalumab in a real-world setting, and propensity-score matching was applied to a historical cohort. They clearly showed significant improvements in local–regional control, PFS and OS in response to durvalumab [35]. This study confirmed the positive effect of durvalumab in the real world. Furthermore, RP and lung dose were found to be significant factors. Because RP also had a relationship with the size of the PTV, the prognostic value of the PTV might be strengthened by immune checkpoint inhibitors. In fact, a larger PTV had already related to significantly shorter PFS and extracranial DMFS after chemoradiotherapy and concurrent and/or sequential immune checkpoint inhibitors [36]. In this study, grade 2 or higher RP on DMFS and lung V20 on PFS were significant prognostic factors. These results suggest that the effect of durvalumab is so profound that factors that discourage treatment with durvalumab might emerge as new factors.

There are several limitations in the present study. This study was a retrospective single-institute study. Due to its retrospective nature, there will be bias and confounding, especially confounding by indication for the administration of durvalumab. The number of patients was relatively small; therefore, variable selection was used to perform MVA. In variable selection, traditional approach of stepwise selection was used, but the modern variable selection approach would be better. A prospective trial with randomization before the start of chemoradiotherapy would be desirable.

## Conclusions

In conclusion, patients treated with durvalumab following IMRT had significantly lower HRs for DMFS, PFS and OS than patients who did not receive durvalumab. In order to initiate and continue durvalumab treatment, the incidence of grade 2 or higher RP should be minimized. Grade 2 or higher RP had a significant correlation with a higher HR of DMFS and a nonsignificant trend for a higher HR of OS. A higher lung V20 had a significant correlation with a higher HR of PFS. Comprehensively, it is important to reduce the irradiated lung dose to decrease the development of grade 2 or higher RP after IMRT.

## Data Availability

The datasets generated and/or analysed during the current study are not publicly available because they contain materials from unpublished manuscripts but are available from the corresponding author upon reasonable request.

## Abbreviations

LA-NSCLC: Locally advanced non-small cell lung cancer
DMFS: Distant metastasis-free survival
PFS: Progression-free survival
OS: Overall survival
RP: Radiation pneumonitis
IMRT: Intensity-modulated radiotherapy
VMAT: Volumetric modulated arc therapy
RECIST: Response Evaluation Criteria in Solid Tumours
CTCAE v5.0: Common Terminology Criteria for Adverse Events version 5.0
GTV: Gross tumour volume
CTV: Clinical target volume
PTV: Planning target volume
V20: Percentage of total lung volume exceeding 20 Gy
V5: Percentage of total lung volume exceeding 5 Gy
UVA: Univariate analyses
MVA: Multivariate analyses
